# The synergistic effect of inflammation and metabolic syndrome on intraocular pressure: A cross-sectional study

**DOI:** 10.1097/MD.0000000000007851

**Published:** 2017-09-08

**Authors:** I-Te Lee, Jun-Sing Wang, Chia-Po Fu, Chia-Jen Chang, Wen-Jane Lee, Shih-Yi Lin, Wayne Huey-Herng Sheu

**Affiliations:** a Division of Endocrinology and Metabolism, Department of Internal Medicine, Taichung Veterans General Hospital, Taichung; b School of Medicine, National Yang-Ming University, Taipei; c School of Medicine, Chung Shan Medical University, Taichung; d Department of Ophthalmology, Taichung Veterans General Hospital, Taichung; e Department of Optometry, Central Taiwan University of Science and Technology, Taichung; f Department of Medical Research, Taichung Veterans General Hospital, Taichung; g Center for Geriatrics and Gerontology, Taichung Veterans General Hospital, Taichung, Taiwan.

**Keywords:** C-reactive protein, inflammation, intraocular pressure, metabolic syndrome

## Abstract

Intraocular pressure is associated with metabolic syndrome. C-reactive protein (CRP) is associated with cardiovascular disease, irrespective of the presence of metabolic syndrome. In this study, we examined the synergistic effect of CRP and metabolic syndrome on intraocular pressure.

A total of 1041 subjects were included for data analyses in this cross-sectional study. Intraocular pressure was measured using a noncontact tonometer, and serum CRP levels were measured using a commercially available kit.

The intraocular pressure was significantly higher in the subjects with metabolic syndrome than in those without (14.1 ± 3.0 vs 13.4 ± 3.0 mm Hg, *P* = .002). Furthermore, intraocular pressures significantly increased according to CRP tertiles (13.1 ± 3.0, 13.7 ± 3.0, and 13.8 ± 3.0 mm Hg from the lowest to highest tertile of CRP, respectively; *P* = .002). The highest intraocular pressure was observed in subjects with metabolic syndrome in the highest CRP tertile (*P* value for trend < .001). Multivariate linear regression analysis revealed that the influence of CRP was independent of metabolic syndrome and that high CRP levels were significantly associated with high intraocular pressure (95% confidence interval: 0.080–1.297, *P* = .027).

In conclusion, systemic inflammation, reflected by serum CRP levels, is associated with high intraocular pressure in subjects with and without metabolic syndrome.

## 1 Introduction

Open-angle glaucoma is one of the major causes of nontraumatic blindness in adults.^[1,2]^ The prevalence of glaucoma is increasing and has become a heavy health burden worldwide.^[3,4]^ High intraocular pressure is a predictor of open-angle glaucoma.^[5–7]^ Therefore, intraocular pressure screening is important in clinical practice.^[8]^

Intraocular pressure is dependent on the balance between the secretion and outflow of aqueous humor.^[9]^ An elevated intraocular pressure can result from a decrease in outflow secondary to an increase in resistance through the trabecular meshwork.^[10]^ Inflammation may induce matrix metalloproteinases dysfunction and alter the extracellular matrix (ECM) components.^[11]^ Therefore, chronic inflammation has the potential to increase the intraocular pressure.^[12]^

Prior reports have shown that intraocular pressure may be associated with cardiovascular risk factors.^[13,14]^ Metabolic syndrome (MetS) is a cluster of cardiovascular risks, and high intraocular pressure is observed in subjects with MetS.^[14–16]^ C-reactive protein (CRP), a novel biomarker for systemic inflammation, has been shown to be a predictor of a cardiovascular disease in subjects with and without MetS.^[17]^ We hypothesized that chronic inflammation, reflected by serum CRP levels, has a synergistic effect on elevating intraocular pressure in subjects with MetS. Therefore, we conducted this observational, cross-sectional study to examine the relationship between serum CRP and intraocular pressure in subjects with and without MetS.

## 2 Methods

### 2.1 Study subjects

This cross-sectional study was conducted at the Taichung Veterans General Hospital. Adult participants undergoing a physical check-up between April 2011 and June 2014 were enrolled. The exclusion criteria were as follows: end-stage renal disease with renal replacement therapy, acute or chronic infectious diseases, intraocular pressure >21 mm Hg, or history of any ocular surgery. The study complies with the Declaration of Helsinki, and the research protocol has been approved by the Institutional Review Board of the Taichung Veterans General Hospital. Written informed consent was obtained from all participants before the study procedure.

Based on the modified criteria of Third Report of the National Cholesterol Education Program, the components of MetS are defined as follows: waist circumference >90 cm in men or >80 cm in women, blood pressure (BP) ≥130/85 mm Hg or use of antihypertensive medications, triglycerides ≥150 mg/dL (1.7 mmol/L), high-density lipoprotein (HDL) cholesterol <40 mg/dL (1.0 mmol/L) in men or <50 mg/dL (1.3 mmol/L) in women, and fasting glucose ≥100 mg/dL (5.6 mmol/L) or use of antidiabetic medications.^[18]^ The diagnosis of MetS is indicated when 3 or more of these components are present.

### 2.2 Biochemical and intraocular pressure measurements

Blood samples were collected in the morning after overnight fasting for measurements of glucose, lipoprotein profiles, liver enzymes, creatinine, and CRP. Glucose, creatinine, liver enzymes, triglyceride, and cholesterol concentrations were measured using Beckman Coulter commercial kits (Fullerton, CA). HDL cholesterol level was measured using a Roche Diagnostics commercial kit (Mannheim, Germany). Levels of CRP were quantified using an immunochemical assay of purified Duck IgY (ΔFc) antibodies (Good Biotech Corp., Taichung, Taiwan). The Modification of Diet in Renal Disease Study equation was used to calculate the estimated glomerular filtration rate (eGFR) by 186 × [serum creatinine (mg/dL)]^−1.154^ × [age (year)]^−0.203^ mL/min/1.73 m^2^ (multiplied by 0.742 for female subjects).^[19]^

The intraocular pressure was measured using a noncontact tonometer (Canon TX-F, Canon, Tokyo, Japan) that obtained measurements using a soft air puff. Measurements were taken in the morning after overnight fasting, with subjects in the sitting position while they pressed their forehead and chin firmly into instrument forehead and chin rests. Three measurements were obtained from each eye and the average of these measurements was recorded as the intraocular pressure. All measurements were obtained by a trained examiner. The subjects were excluded if a retinopathy, glaucoma, or optic nerve disease was diagnosed upon subsequent examination by the ophthalmologist.

### 2.3 Statistical analyses

Continuous data are presented as mean ± standard deviation and categorical data are presented as numbers (percentage). Chi-square tests were used to determine the significance of differences in categorical variables. Independent-sample *t* tests were used to determine the significance of differences between groups in continuous variables. One-way analysis of variance tests were used to compare differences in intraocular pressure among more than 2 subject groups. Levels of CRP were divided into tertiles (first tertile = lowest third and third tertile = highest third). A test for trends of intraocular pressure across MetS and CRP tertile groups was then performed. Multivariate linear regression analysis was used to identify factors associated with intraocular pressure. There was a good positive correlation in intraocular pressure between right and left eyes (*r* = 0.755, *P* < .001) based on Pearson correlation, and only left eye data were included for analyses in the present study. Statistical analyses were performed using SPSS statistical software (version 22, IBM, Armonk, New York).

## 3 Results

A total of 1061 adults were enrolled in this study. Of these, 1041 subjects satisfied all enrollment criteria. The mean age was 48 ± 11 years, and 694 subjects (66.7%) were men. There were 225 subjects with MetS and 816 without MetS. Subjects with MetS were significantly older than those without MetS (52 ± 10 vs 47 ± 12 years, *P* < .001). Subjects with MetS were more likely to be male dominant than those without MetS (80.4% vs 62.9%, *P* < .001). Intraocular pressure was higher in subjects with MetS (14.1 ± 3.0 mm Hg) than in those without MetS (13.4 ± 3.0 mm Hg, *P* = .002). CRP levels were also higher in subjects with MetS (2.3 ± 3.3 mg/dL) than in those without MetS (1.4 ± 2.9 mg/dL, *P* < .001). Furthermore, body mass index (BMI) and waist circumference were higher in subjects with MetS than in those without MetS (both *P* < .001). Both systolic and diastolic BPs were higher in subjects with MetS than in those without MetS (both *P* < .001). Higher fasting levels of serum triglycerides and lower levels of HDL cholesterol were detected in subjects with MetS than in those without MetS (both *P* < .001). Fasting glucose and the proportion of diabetes were significantly higher in subjects with MetS than in those without MetS (both *P* < .001). Liver enzymes including aspartate aminotransferase and alanine aminotransferase were higher in subjects with MetS than in those without MetS (both *P* < .001). A lower eGFR was detected in subjects with MetS than in those without MetS (*P* < .001). However, the proportion of current smoker was not significantly different between subjects with and without MetS (*P* = .236) (Table 1).

**Table 1:**
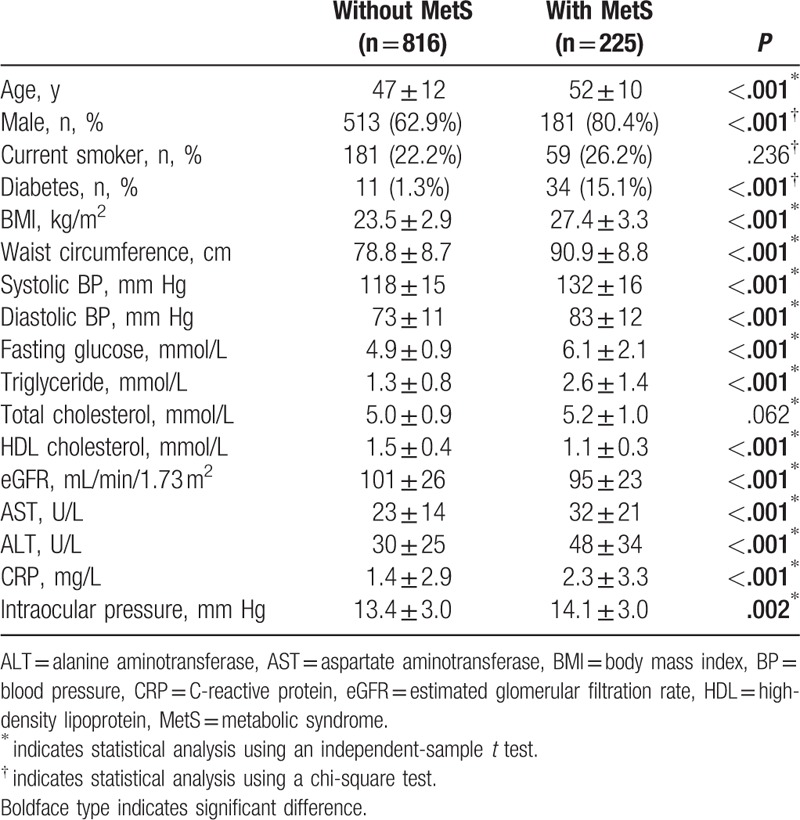
Anthropometric and biochemical data of subjects with and without MetS.

As shown in Table 2, higher intraocular pressure was significantly associated with young age (*P* = .010), male sex (*P* = .006), current smoking habit (*P* = .033), central obesity (*P* = .002), hypertension (*P* < .001), hypertriglyceridemia (*P* = .019), and higher CRP (*P* = .002). However, intraocular pressure was not significantly associated with HDL cholesterol (*P* = .500) or fasting glucose (*P* = .155) levels.

**Table 2:**
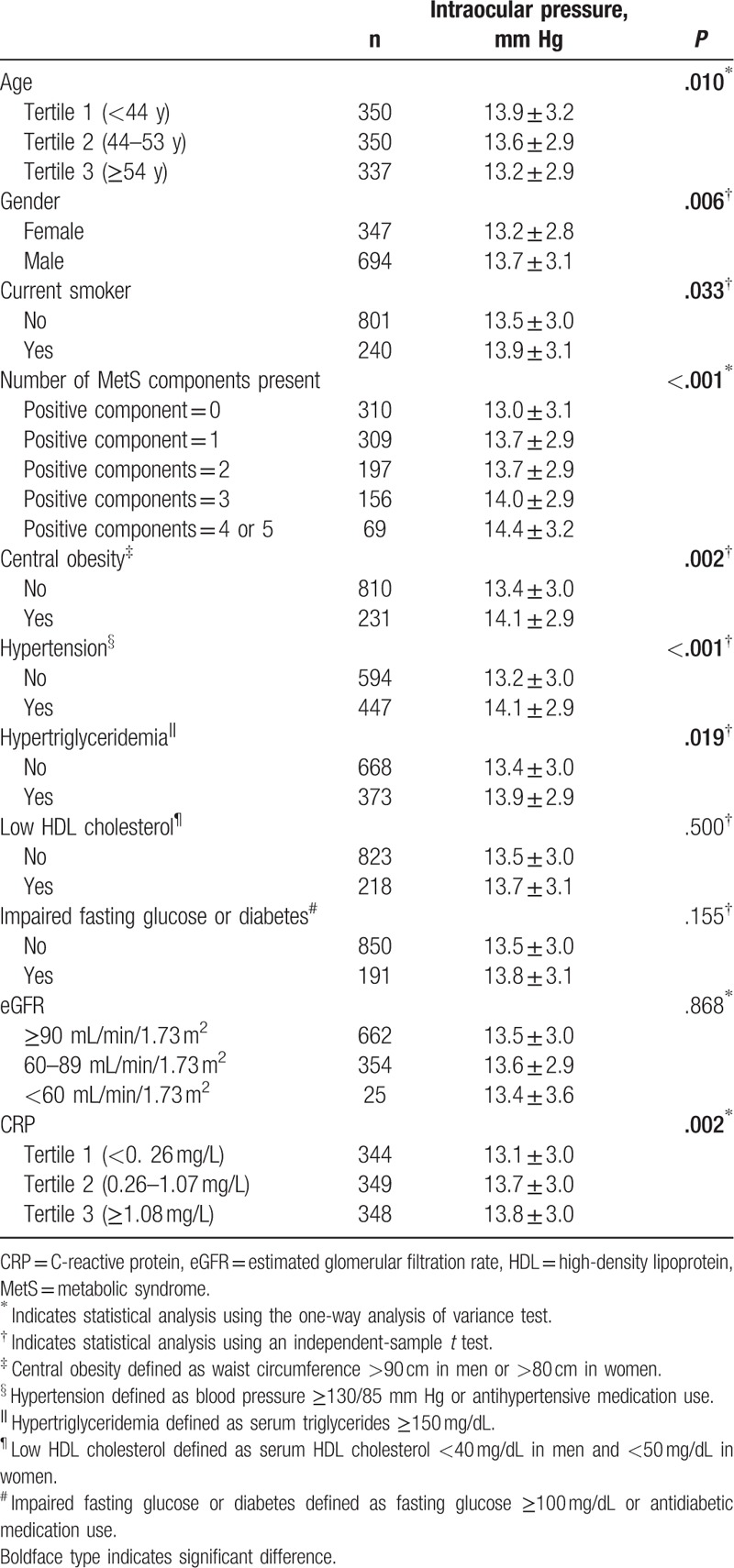
Effects of subject characteristics on intraocular pressure.

**Figure 1:**
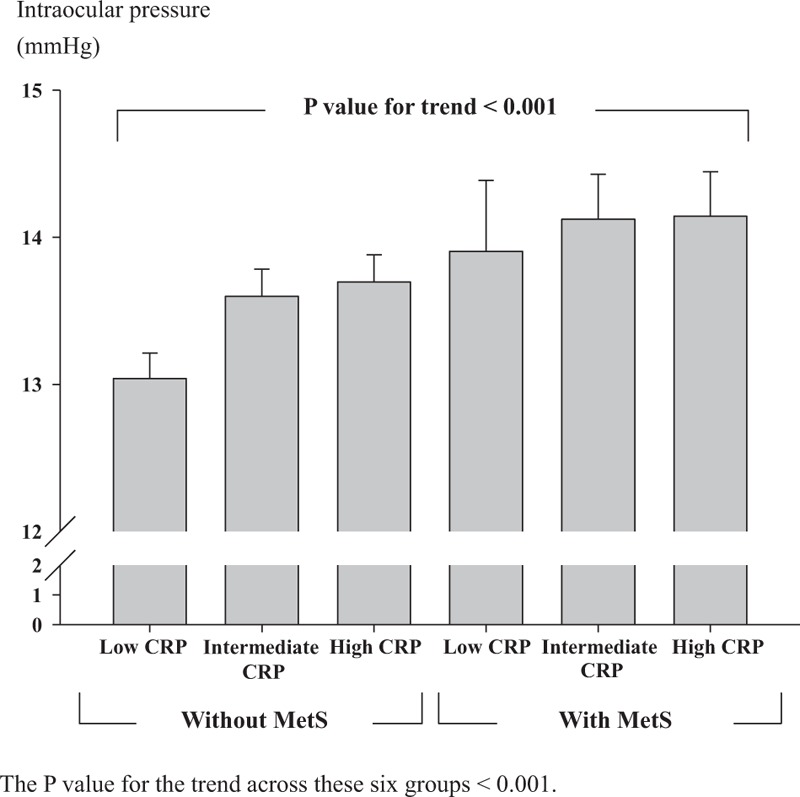
Mean intraocular pressure in each CRP tertile in subjects with and without MetS. The *P* value for the trend across these 6 groups is <.001. CRP = C-reactive protein, MetS = metabolic syndrome.

Intraocular pressures in different CRP tertiles of the subjects with MetS and without MetS are shown in Figure 1. There was a positive trend in intraocular pressure to CRP and MetS (*P* value for the test of trend across these 6 groups <.001). Linear regression analysis also revealed that age (95% confidence interval [CI]: −0.475 to −0.136, *P* < .001), presence of MetS (95% CI: 0.218–1.123, *P* = .004), and CRP (95% CI: 0.080–1.297, *P* = .027) independently influenced intraocular pressure (Table 3).

**Table 3:**
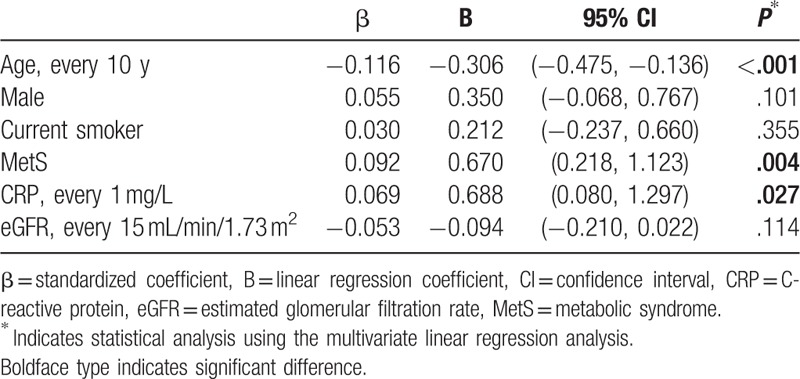
Linear regression analysis showing the effect of risk factors on intraocular pressure.

## 4 Discussion

In the present study, the intraocular pressure was higher in subjects with MetS than in those without MetS. An interesting finding in our analyses is that intraocular pressure showed a positive association with serum CRP levels. Although the pathological mechanisms that link systemic inflammation and intraocular pressure are not well understood,^[12]^ it has been reported that glaucoma is associated with endothelial dysfunction, as reflected by high levels of circulating endothelin-1 and vasoconstriction.^[20,21]^ Endothelium-associated vascular dysregulation may be involved in the inflammatory process.^[22]^ Furthermore, the collagen beams of the trabecular meshwork are covered by endothelial cells, and the ECM fills the spaces between the beams.^[23]^ Mucopolysaccharides from endothelial cells are associated with macrophage function, and contribute to the components of the ECM. An alteration in the ECM may cause an increased resistance in the trabecular meshwork and a consequent decrease in outflow.^[24,25]^ Our results support the idea that systemic inflammation may result in an increase in intraocular pressure. In line with our finding, Cellini et al^[22]^ previously reported a significant association between systemic endothelial dysfunction and open-angle glaucoma.

Although the association between intraocular pressure and cardiovascular risk factors has been reported in several studies,^[14,15,26]^ the results have been inconsistent. It has been reported that intraocular pressure decreased by age of 40 years in cross-sectional data.^[27–29]^ In a longitudinal assessment, the average annual change of intraocular pressure was found to be −0.065 mm in a Korean population.^[29]^ In contrast, intraocular pressure increased with age in northern Sweden in a prospective follow-up study of 21 years.^[30]^ In a Japanese study, intraocular pressure was found to be inversely associated with age in the cross-sectional assessment, but positively associated during the follow-up.^[31]^ This inconsistency between cross-sectional and longitudinal assessments might result from differences in lifestyle in recent decades. In the present study, higher intraocular pressure was significantly associated with younger age, which is consistent with a recent report in a Taiwanese population.^[15]^ Because lifestyle is associated with intraocular pressure,^[32]^ we could not exclude the possibility of poor health habits in younger Taiwanese.

Previous studies in the United States and northern Sweden reported higher intraocular pressures in women than in men,^[30,33,34]^ but the converse was reported in some studies of Asian populations.^[35–38]^ In the present study, despite a higher intraocular pressure in male subjects, gender was not an independent factor for intraocular pressure after adjusting for cardiovascular risk factors. No significant difference in intraocular pressure between genders was reported in previous Taiwanese and Korean investigations.^[15,39]^ In the Beaver Dam Eye Study, gender was still not an independent factor for intraocular pressure, despite a higher pressure in women than in men.^[34]^

There were cumulative effects of MetS components on increasing intraocular pressure.^[39,40]^ However, the effect of each MetS component was not the same.^[35,41]^ Of the components of MetS, only central obesity, hypertension, and hypertriglyceridemia were significantly associated with higher intraocular pressure in the present study. Obesity may be accompanied by excessive adipose tissue of the intraorbital region, increasing the resistance to aqueous outflow.^[42]^ In addition, obesity is associated with endothelial dysfunction, which is a cause of open-angle glaucoma.^[22]^ BMI as well as waist circumference have been reported to be associated with intraocular pressure in several studies,^[13,33,34,43]^ and the association was also observed in prospective investigations.^[37,38,44]^

High systemic arterial pressure elevates the ciliary artery pressure and increases the aqueous fluid filtration which can induce an increase in intraocular pressure.^[45]^ Furthermore, hypertension can cause an increased blood volume in the ciliary body and decrease aqueous outflow.^[46]^ In the Beijing Eye Study, both systolic and diastolic BPs were positively correlated with intraocular pressure after exclusion of intraocular pressure >21 mm Hg.^[47]^ In a large Japanese investigation, systemic BP and BMI were both associated with intraocular pressure changes.^[31]^ Recently, changes in intraocular pressure were also reported to be positively associated with changes in either systemic BP or BMI in longitudinal studies.^[37,48]^ Since intraorbital adipose tissue increases the episcleral venous pressure and decreases the outflow facility, hypertriglyceridemia, associated with an excessive fat status, is a risk factor of high intraocular pressure.^[36,43]^ In a longitudinal follow-up study, changes in intraocular pressure were reported to be positively correlated with increases in serum triglycerides in a Japanese population.^[49]^

In the present study, the intraocular pressure was not significantly different between subjects with high HDL and low HDL levels. In line with our findings, circulating HDL cholesterol levels were not associated with intraocular pressure in the majority of investigations for MetS.^[15,35,41,43]^ Furthermore, there was no significant difference in intraocular pressure between subjects with normal fasting glucose and higher fasting glucose in the present study. It is notable that diabetes and insulin resistance are well documented to be associated with increased intraocular pressure,^[40,50]^ and chronic hyperglycemia could be a predictor of high intraocular pressure in diabetic patients.^[51]^ Nevertheless, it was reported that hyperglycemia with increased circulating osmolality might reduce intraocular pressure,^[52]^ and these effects might have resulted in the null-hypothesis finding in the present study.

Smoking was reported to be associated with increased intraocular pressure,^[53]^ but was not associated with open-angle glaucoma in a large investigation among subjects from the Nurses’ Health Study and Health Professionals Follow-Up Study.^[54]^ In the present study, the intraocular pressure was higher in current smokers than in others. However, smoking was not an independent risk factor for intraocular pressure after adjusting for cardiovascular risk factors.

Goldmann applanation tonometry is the gold standard for measuring intraocular pressure. However, noncontact tonometry is easy to use and eliminate the potential for contact infection.^[55]^ Furthermore, several studies have shown that air puff noncontact tonometry measurements of intraocular pressure are reliable and in good agreement with Goldmann applanation tonometry measurements.^[56,57]^ In line with our findings, MetS is significantly associated with raising intraocular pressure detected using Goldmann applanation tonometry in the Korean National Health and Nutrition Examination Survey.^[43]^

There are several limitations in the present study. First, we did not assess the mechanism underlying the association between intraocular pressure and systemic inflammation. Second, we only assessed intraocular pressure, and did not directly investigate open-angle glaucoma. Although there is a synergistic effect of MetS and CRP on intraocular pressure, neither atherosclerotic risks nor CRP could accurately predict open-angle glaucoma in a prospective 6.5-year follow-up study.^[58]^ Therefore, further studies to investigate the link between systemic inflammation and open-angle glaucoma are warranted.

In conclusion, our findings suggest that systemic inflammation might be a risk factor for high intraocular pressure, and that there is a synergistic effect of CRP and MetS on raising intraocular pressure.
